# Kras Gene Mutation and RASSF1A, FHIT and MGMT Gene Promoter Hypermethylation: Indicators of Tumor Staging and Metastasis in Adenocarcinomatous Sporadic Colorectal Cancer in Indian Population

**DOI:** 10.1371/journal.pone.0060142

**Published:** 2013-04-03

**Authors:** Rupal Sinha, Showket Hussain, Ravi Mehrotra, R. Suresh Kumar, Kapil Kumar, Pankaj Pande, Dinesh Chandra Doval, Seemi Farhat Basir, Mausumi Bharadwaj

**Affiliations:** 1 Department of Research, Rajiv Gandhi Cancer Institute and Research Centre, Delhi, India; 2 Division of Molecular Genetics and Biochemistry, Institute of Cytology and Preventive Oncology, Noida, India; 3 Division of Cytopathology, Institute of Cytology and Preventive Oncology, Noida, India; 4 Department of Surgical Oncology, Rajiv Gandhi Cancer Institute and Research Centre, Delhi, India; 5 Department of Medical Oncology, Rajiv Gandhi Cancer Institute and Research Centre, Delhi, India; 6 Department of Biosciences, Jamia Millia Islamia, New Delhi, India; Howard University, United States of America

## Abstract

**Objective:**

Colorectal cancer (CRC) development involves underlying modifications at genetic/epigenetic level. This study evaluated the role of Kras gene mutation and RASSF1A, FHIT and MGMT gene promoter hypermethylation together/independently in sporadic CRC in Indian population and correlation with clinicopathological variables of the disease.

**Methods:**

One hundred and twenty four consecutive surgically resected tissues (62 tumor and equal number of normal adjacent controls) of primary sporadic CRC were included and patient details including demographic characteristics, lifestyle/food or drinking habits, clinical and histopathological profiles were recorded. Polymerase chain reaction - Restriction fragment length polymorphism and direct sequencing for Kras gene mutation and Methylation Specific-PCR for RASSF1A, FHIT and MGMT genes was performed.

**Results:**

Kras gene mutation at codon 12 & 13 and methylated RASSF1A, FHIT and MGMT gene was observed in 47%, 19%, 47%, 37% and 47% cases, respectively. Alcohol intake and smoking were significantly associated with presence of Kras mutation (codon 12) and MGMT methylation (p-value <0.049). Tumor stage and metastasis correlated with presence of mutant Kras codon 12 (p-values 0.018, 0.044) and methylated RASSF1A (p-values 0.034, 0.044), FHIT (p-values 0.001, 0.047) and MGMT (p-values 0.018, 0.044) genes. Combinatorial effect of gene mutation/methylation was also observed (p-value <0.025). Overall, tumor stage 3, moderately differentiated tumors, presence of lymphatic invasion and absence of metastasis was more frequently observed in tumors with mutated Kras and/or methylated RASSF1A, FHIT and MGMT genes.

**Conclusion:**

Synergistic interrelationship between these genes in sporadic CRC may be used as diagnostic/prognostic markers in assessing the overall pathological status of CRC.

## Introduction

Colorectal cancer (CRC) is the third most common cancer in men (663000 cases) and the second in women (571000 cases) with about 608000 deaths estimated worldwide, making it the fourth most common cause of death from cancer. In India, the estimated incidence and mortality from CRC is 36476 cases and 25690 cases, respectively [1]. CRC rates are about 2 to 5 times higher in the developed countries in comparison to the developing countries which may be attributable to a range of variations in a disparate set of risk factors and diagnostic practices [2], [3]. Lifestyle related predisposing modifiable risk factors for CRC include physical inactivity, overweight and obesity, red and processed meat consumption, smoking and excessive alcohol consumption [3].

CRC is a heterogeneous disease with complex etiology and may be caused, in part, by genetic and epigenetic alterations which may act synergistically and transform the epithelial cells into adenocarcinomas [4]. A genetic alteration that occurs in adenomas (10%) as well as carcinomas (40%) in colon and rectal cancer is the oncogenic activation of the Kras gene by mutations [5]. The Kras proto-oncogene encodes a protein (p21-ras) belonging to the family of GTP/GDP-binding proteins with GTPase activity and is involved in the transduction of mutagenic signals [6].

Epigenetic silencing of genes also plays an important role in the inactivation of tumor suppressor genes in carcinogenesis [7]. Aberrant DNA methylation in the CpG islands at the promoter region begins early in tumorigenesis and is an important epigenetic mechanism underlying the inactivation of tumor suppressor genes [8]. RASSF1 gene is a putative tumor suppressor gene acting at G1/S phase of cell-cycle progression [9]. Gene silencing as a result of aberrant promoter methylation in RASSF1A may be an important causative event in tumorigenesis and has been recognized as an alternative marker to downregulate Ras pathway [10].

The human FHIT gene, a tumor suppressor gene, is a member of the histidine triad gene family [11]. Methylation of the FHIT gene has been observed in several solid tumors and an abridged or complete loss of expression of FHIT protein has been shown to be due to gene methylation leading to its transcriptional inactivation and disease progression [12].

O^6^-methylguanine-DNA methyltransferase (MGMT) is a DNA repair enzyme removing alkyl groups from the O^6^ position of guanine [13]. MGMT promoter hypermethylation and epigenetic silencing trigger often occur as early events in carcinogenesis [14].

The present study was thus conducted to study the role of genetic mutation in the Kras gene and epigenetic modification in RASSF1A, FHIT and MGMT genes either together or independently in adenocarcinomatous sporadic CRC in the Indian population and their correlation with the clinicopathological variables of CRC.

## Materials and Methods

### Patients and Tissue Specimens

A total of 124 consecutive surgically resected fresh tissue specimens comprising of 62 tumor tissues and 62 adjacent normal control regions (normal non tumorous healthy mucosa at >2 cm distance from the tumor) [15] of primary sporadic CRC were collected for analysis in the present study. Only those cases fulfilling our inclusion criteria such as consecutive cases with a primary diagnosis of colorectal cancer undergoing upfront surgery were taken up for the purpose of this study. None of these patients had received any pre-operative treatment in the form of radiation or chemotherapy. All the tissue samples were collected within 10 minutes of resection. Tissue samples were divided into two parts; one part was sent for histopathological diagnosis and staging and the other half was stored at −80°C for molecular analysis. Histopathological grades and clinical staging were evaluated according to the standard criteria [16] by two independent pathologists with 5 cases graded as well differentiated adenocarcinoma, 41 as moderately differentiated adenocarcinoma, 15 as poorly differentiated adenocarcinoma and 1 patient as undifferentiated adenocarcinoma whereas 8, 25, 25 and 4 patients were scored as stage 1, 2, 3 and 4, respectively. Only histopathologically confirmed cases were included for further molecular analysis. Details of each patient related to their demographic profile, habits, signs and symptoms, personal history, investigations, tumor profile including metastasis and histopathology reports, further treatment and follow up information was also recorded. In terms of dietary habits, spicy food was classified as the use of spices especially hot spices and chilies in the diet in every meal regularly (3–5 gms per meal), non vegetarian diet was considered as the intake of red meat (at least thrice per week) and fatty diet was judged upon intake of high fat diet of any type with increased use of cooking oils in every meal (>10 ml mustard oil per meal). All the patients enrolled in the study were chronic alcoholics (average use of alcohol 100–150 ml per day at least 3–4 times per week), smokers (at least one pack of 10 cigarettes per day) and tobacco chewers (4–6 packs per day). The study was approved by the ethical committees of the participating institutions and prior written informed consent was taken from the patients before enrolment in the study. The study was carried out in accordance with the principles of Helsinki Declaration [17].

### DNA Extraction

High molecular-weight genomic DNA was extracted from ∼300 mg of tumor and normal adjacent tissue specimens by the standard method with proteinase K digestion followed by phenol-chloroform extraction [18]. DNA quantity/quality was checked spectrophotometrically (Nanodrop ND-1000 version 3.6.0, Thermoscientific, Willington DE USA)/1% agarose gel electrophoresis and stored for further use at −20°C.

### Kras Mutation Analysis

DNA was amplified in a 25 µl reaction mixture containing 25 pmoles of both forward and reverse primers, 25 mM each dNTP, and 0.2 U Taq DNA polymerase. The primer sequences have been described elsewhere [19]. The oligonucleotide primers were synthesized in an automated Applied Biosystems DNA synthesizer (Model 381A; Applied Bio-systems, Foster City, CA, USA) using the phosphoramidite method and purified in high performance liquid chromatography (HPLC). Further, restriction fragment length polymorphism analysis was done with MvaI and BglI to check the presence of mutations in codon 12 and 13 of Kras gene. The digestion products were then visualized by ethidium bromide staining under UV light after electrophoresis on 4% agarose gel. Results were confirmed by sequencing.

### Methylation Specific Polymerase Chain Reaction (MSP)

Genomic DNA was modified using the EZ DNA Methylation Gold Kit (Pro Lab Marketing, Delhi, India) as per the instructions available followed by PCR with methylated and unmethylated specific primers to define the methylation patterns. The primer sequences of each gene promoter for both methylated and unmethylated forms have been described previously [7], [20]. Hot start PCR was performed and the products were electrophoresed on a 2.5% agarose gel and visualized under UV illumination after ethidium bromide staining.

### Statistical Analysis

All statistical analyses were performed by the standard methods using SPSS computer software (Version 16, SPSS Inc, Chicago, IL, USA). Fischer's Exact Test or Chi Square Tests were used as applicable. The findings were considered statistically significant at p values of <0.05. Overall survival (OS) was calculated by the Kaplan Meier survival method [21].

## Results

### Overall Analysis Based on Demographic & Clinical Profile and Survival of CRC Patients

Of the 62 cases analyzed, tumor was located in the colon, rectum and rectosigmoid regions in 66%, 24% and 10% cases, respectively. Majority of the patients (76%) were males. The mean age of the cases included in the study was 55 years at the time of diagnosis. Co morbidities including diabetes, hypertension, tuberculosis, etc were reported in 27% cases and 16% cases had a family history of cancer while none of the cases had a family history of CRC. Loss of weight was the most common sign (63%, mean duration 6 months), followed by abdominal pain (57%, mean duration 1 year) and bleeding per rectum (50%, mean duration 3 months). Amongst the lifestyle factors, high intake of fatty diet was most commonly observed (52%) followed by non-vegetarian diet (48%) and food rich in spices (47%) (Table 1). Alcohol intake and smoking habits were observed in 45% and 42% patients, respectively in our study. The association was further evident in the case of male patients where 60% and 53% patients had the above habits (p-values <0.001 & 0.001, respectively). The biochemical profile of all the patients is included in Table S1. The overall survival of the patients was 94% at 1 year with a median follow up of 2 months (range 1–19 months).

**Table 1:** Demographic profile and follow up of 62 patients with sporadic CRC.

Characteristic	N (%)
Mean age at diagnosis	55.4+13.6 years
Males : Females	47 (75.8) : 15 (24.2)
Co morbidities*	17 (27.4)
Family history of cancer	10 (16.1)
***Signs & Symptoms***	
Loss of weight	39 (62.9)
Abdominal pain	35 (56.5)
Bleeding per rectum	31 (50)
Constipation	30 (48.4)
Fatigue	28 (45.2)
Obstruction features	24 (38.7)
Loss of appetite	24 (38.7)
Back pain	21 (33.9)
Diarrhea	16 (25.8)
Nausea & Vomiting	9 (14.5)
Difficulty in micturition	6 (9.7)
***Lifestyle factors***	
Fatty diet	32 (51.6)
Non-vegetarian diet	30 (48.4)
Spicy food	29 (46.8)
Alcohol intake	28 (45.2)
Smoking	26 (41.9)
Lack of physical exercise	21 (33.9)
Irregular intake of fruits	18 (29)
Tobacco chewing	14 (22.6)
***Follow up***	
Overall survival	94% at 1 year
Status - Dead	1 (1.6)
Median follow up	2 months(Range 1–19 months)

* Co morbidities include hypertension, diabetes, heart diseases and tuberculosis.

### Analysis of Kras Mutations at Codon 12 and 13 in CRC

Considering the important role of RAS proteins, particularly Kras in carcinogenesis, Kras gene mutation was analysed and observed in 47% (29/62) cases at codon 12 and in 19% (12/62) cases at codon 13 in the tumor region. The mutation was also observed in 29% and 18% adjacent control regions at codon 12 and 13, respectively. Alcohol intake, smoking and intake of spicy food was observed more frequently in patients with mutant Kras codon 12 as compared to those with wild type Kras (59%, 69%, 55% vs. 33%, 18% and 39%, respectively) and was statistically significant with respect to alcohol consumption and smoking habits (p-values <0.047, 0.001) (Table 2). Most of the cases with Kras gene mutation codon 12 were characteristic of tumor stage 3 (52%) and histologically moderately differentiated grade (66%). Tumor stage and metastasis were also found to be significantly associated with the presence of mutant Kras codon 12 (p-values 0.018, 0.044). However, association of tumor grade and lymphatic invasion with mutant Kras was not statistically significant (Table 3).

**Table 2:** Lifestyle factors of 62 patients with sporadic CRC.

Characteristic	Kras gene codon 12			Kras gene codon 13			RASSF1A gene			FHIT gene			MGMT gene		
	Wild type n = 33 (%)	Mutant n = 29 (%)	p-value	Wild type n = 50 (%)	Mutant n = 12 (%)	p-value	UM* n = 33(%)	M* n = 29(%)	p-value	UM* n = 39(%)	M* n = 23(%)	p-value	UM* n = 33(%)	M* n = 29(%)	p-value
Alcohol intake	11 (33.3)	17 (58.6)	**0.046**	22 (44)	6 (50)	0.708	12 (36.4)	16 (55.2)	0.138	17 (43.6)	11 (47.8)	0.746	10 (30.3)	18 (62.1)	**0.012**
Tobacco chewing	9 (27.3)	5 (17.2)	0.346	13 (26)	1 (8.3)	0.189	8 (24.2)	6 (20.7)	0.739	6 (15.4)	8 (34.8)	0.078	7 (21.2)	7 (24.1)	0.783
Smoking	6 (18.2)	20 (68.9)	**<0.001**	18 (36)	8 (66.7)	0.053	10 (30.3)	16 (55.2)	**0.048**	18 (46.2)	8 (34.8)	0.381	7 (21.2)	19 (65.5)	**<0.001**
Fatty diet	18 (54.5)	14 (48.3)	0.622	25 (50)	7 (58.3)	0.604	16 (48.5)	16 (55.2)	0.599	20 (51.3)	12 (52.2)	0.946	18 (54.5)	14 (48.3)	0.622
Irregular intakeof fruits	8 (24.2)	10 (34.5)	0.375	15 (30)	3 (25)	0.732	12 (36.4)	6 (20.7)	0.175	10 (25.6)	8 (34.8)	0.444	8 (24.2)	10 (34.5)	0.375
Spicy food	13 (39.4)	16 (55.2)	0.214	24 (48)	5 (41.7)	0.693	15 (45.5)	14 (48.3)	0.824	18 (46.2)	11 (47.8)	0.899	14 (42.4)	15 (51.7)	0.464
Non-vegetariandiet	16 (48.5)	14 (48.3)	0.987	27 (54)	3 (25)	0.071	17 (51.5)	13 (44.8)	0.599	16 (41)	14 (60.9)	0.131	15 (45.5)	15 (51.7)	0.622
Lack of physicalexercise	12 (36.4)	9 (31)	0.658	18 (36)	0 (0)	0.470	14 (42.4)	7 (24.1)	0.129	11 (28.2)	10 (43.5)	0.220	10 (30.3)	11 (37.9)	0.527

* UM, unmethylated; M, methylated.

p value <0.05 was taken as significant.

**Table 3:** Tumor profile of 62 patients with sporadic CRC.

	Kras gene codon 12			Kras gene codon 13			RASSF1A gene			FHIT gene			MGMT gene		
	Wild typen = 33	Variantn = 29	p-value	Wild typen = 50	Variantn = 12	p-value	UM* n = 33	M* n = 29	p-value	UM* n = 39	M* n = 23	p-value	UM* n = 33	M* n = 29	p-value
**Tumor location**															
ColonRectumRectosigmoid	22 (66.6)6 (18.2)5 (15.2)	19 (65.5)9 (31)1 (3.5)	0.198	33 (66)11 (22)6 (12)	8 (66.7)4 (33.3)0 (0)	0.376	18 (54.5)11 (33.3)4 (12.1)	23 (79.3)4 (13.8)2 (6.9	0.116	26 (66.7)10 (25.6)3 (7.7)	15 (65.2)5 (21.7)3 (13.1)	0.770	20 (60.6)9 (27.3)4 (12.1)	21 (72.4)6 (20.7)2 (6.9)	0.595
**Tumor stage**															
1234	6 (18.2)17 (51.5)10 (30.3)0 (0)	2 (6.9)8 (27.6)15 (51.7)4 (13.8)	**0.018**	7 (14)20 (40)21 (42)2 (4)	1 (8.3)5 (41.7)4 (33.3)2 (16.7)	0.418	7 (21.2)14 (42.4)12 (36.4)0 (0)	1 (3.5)11 (37.9)13 (44.8)4 (13.8)	**0.034**	8 (20.5)20 (51.3)9 (23.1)2 (5.1)	0 (0)5 (21.7)16 (69.6)2 (8.7)	**0.001**	5 (15.2)18 (54.5)10 (30.3)0 (0)	3 (10.4)7 (24.1)15 (51.7)4 (13.8)	**0.018**
**Tumor grade** #															
WDMDPDU	1 (3)22 (66.7)10 (30.3)0 (0)	4 (13.8)19 (65.5)5 (17.2)1 (3.5)	0.217	4 (8)34 (68)12 (24)0 (0)	1 (8.3)7 (58.4)3 (25)1 (8.3)	0.230	2 (6.1)24 (72.7)7 (21.2)0 (0)	3 (10.3)17 (58.6)8 (27.6)1 (3.5)	0.529	4 (10.2)26 (66.7)8 (20.5)1 (2.6)	1 (4.4)15 (65.2)7 (30.4)0 (0)	0.613	1 (3)22 (66.7)9 (27.3)1 (3)	4 (13.8)19 (65.5)6 (20.7)0 (0)	0.337
**Lymphatic invasion**															
AbsentPresent	22 (66.7)11 (33.3)	14 (48.3)15 (51.7)	0.143	31 (62)19 (38)	5 (41.7)7 (58.3)	0.200	23 (69.7)10 (30.3)	13 (44.8)16 (55.2)	**0.048**	28 (71.8)11 (28.2)	8 (34.8)15 (65.2)	**0.004**	23 (69.7)10 (30.3)	13 (44.8)6 (55.2)	**0.048**
**Metastasis**															
AbsentPresent	31 (93.9)2 (6.1)	22 (75.9)7 (24.1)	**0.044**	43 (86)7 (14)	10 (83.3)2 (16.7)	0.814	31 (93.9)2 (6.1)	22 (75.9)7 (24.1)	**0.044**	36 (92.3)3 (7.7)	17 (73.9)6 (26.1)	**0.047**	31 (93.9)2 (6.1)	22 (75.9)7 (24.1)	**0.044**
**Status**															
AliveDead	32 (97)1 (3)	29 (100)0 (0)	0.345	49 (98)1 (2)	12 (100)0 (0)	0.621	32 (97)1 (3)	29 (100)0 (0)	0.345	38 (97.4)1 (2.6)	23 (100)0 (0)	0.439	32 (97)1 (3)	29 (100)0 (0)	0.345

* UM, unmethylated; M, methylated;

# WD, well differentiated; MD, moderately differentiated; PD, poorly differentiated; U, undifferentiated.

p value <0.05 was taken as significant.

### Analysis of RASSF1A Promoter Methylation in CRC

Promoter hypermethylation in RASSF1A gene was observed in 47% (29/62) cases of CRC in the tumor region and in 13% of adjacent controls. Amongst the lifestyle factors, the frequency of alcohol consumption, fatty diet and smoking was more in case of patients with methylated RASSF1A as compared to those with unmethylated RASSF1A (55%, 55% and 55% vs. 36%, 49% and 30%, respectively) (Table 2). A significant number of cases with methylated RASSF1A gene had tumor stage 3 (45%) and moderately differentiated grade (59%). Tumor stage, metastasis and lymphatic invasion correlated significantly with the presence of methylated RASSF1A (p-values 0.034, 0.044 and 0.048, respectively), However, there was no association of tumor grade with methylated RASSF1A (Table 3).

### Analysis of FHIT Promoter Methylation in CRC

Methylated FHIT gene was observed in 37% (23/62) cases of CRC. FHIT methylation was also observed in 13% cases in the adjacent control region. Patients with methylated FHIT gene had a higher incidence of non-vegetarian and fatty diet as compared to the unmethylated group (61% and 52% vs. 41% and 51%, respectively). However, no statistical association was observed (Table 2). Majority of the cases with methylated FHIT gene were tumor stage 3 (70%) and moderately differentiated histological grade (65%). Tumor stage, metastasis and presence of lymphatic invasion were significantly linked with the presence of methylated FHIT (p-values 0.001, 0.047 and 0.004, respectively). However, we failed to observe any association between tumor grade and methylated FHIT (Table 3).

### Analysis of MGMT Promoter Methylation in CRC

Methylated MGMT gene was observed in 47% (29/62) cases of CRC in the tumor region and in 13% cases in the adjacent control regions. Also, smoking and alcohol intake was more commonly reported by patients with methylated MGMT gene as compared to the unmethylated group (66% and 62% vs. 21% and 30%, respectively). The correlation was particularly evident and conclusive in the case of alcohol intake and smoking and the presence of MGMT methylation (p-values <0.013, 0.001) (Table 2). Higher number of cases with methylated MGMT gene were tumor stage 3 (52%) and moderately differentiated grade (66%). Tumor stage, metastasis and lymphatic invasion were found to be significantly correlated with the presence of methylated MGMT gene (p-values 0.018, 0.044 and 0.048, respectively). However, association of tumor grade with methylated MGMT gene was not statistically significant (Table 3).

### Combinatorial Effect of Genetic Mutation in Kras Gene and Epigenetic Modification in RASSF1A, FHIT and MGMT Genes in CRC

On comparison of occurrence of mutation/methylation of one gene in the presence of mutation/methylation of another gene, it was observed that the genes had the combined presence of mutation/methylation in the majority of the cases and the results were found to be statistically significant (p-values <0.025, 0.005) (Table 4). Only 15% (9/62) patients did not show the presence of Kras mutation or RASSF1A, FHIT and MGMT methylation. Interestingly, Kras mutation/RASSF1A, FHIT and MGMT methylation were more common in patients with a previous history of alcohol consumption 89% (25/28), tobacco chewing 86% (12/14), smoking 96% (25/26), fatty diet 78% (25/32), irregular intake of fruits 100% (18/18), intake of spicy food 79% (23/29) and non-vegetarian diet 87% (26/30). Also, none of the cases had the presence of mutation/methylation in the adjacent control region only and not in the tumor region. Further, a comparison of the clinical profile with the combinatorial effect of variations in genes in 62 patients with sporadic CRC was performed and it was observed that the clinical features were more commonly observed in patients with increasing number of variation in the genes in terms of genetic mutation in Kras gene and/or promoter hypermethylation in MGMT, FHIT and RASSF1A genes in sporadic CRC (Table 5). Overall, advanced tumor stage (97%), metastatic disease (100%), moderately to poorly differentiated tumors (84%) and presence of lymphatic invasion (92%) were more frequently observed in tumors with mutated Kras (codon 12) and methylated RASSF1A, FHIT and MGMT genes. Also, promoter hypermethylation in RASSF1A, FHIT and MGMT genes correlated significantly with the presence of lymphatic invasion in contrast to mutation in the Kras gene.

**Table 4:** Occurrence of mutation/methylation in one gene in the presence of mutation/methylation in another gene.

	Kras gene codon 12None/Either/Bothp-value	Kras gene codon 13None/Either/Bothp-value	RASSF1A geneNone/Either/Bothp-value	FHIT geneNone/Either/Bothp-value	MGMT geneNone/Either/Bothp-value
**Kras gene codon 12**None/Either/Both p-value	–	31/21/10 **0.005**	22/22/18 **0.024**	23/26/13 0.237	22/22/18 **0.024**
**Kras gene codon 13**None/Either/Both p-value	31/21/10 **0.005**	–	31/21/10 **0.005**	30/29/3 0.334	28/27/7 0.372
**RASSF1A gene**None/Either/Both p-value	22/22/18 **0.024**	31/21/10 **0.005**	–	21/30/11 0.899	20/26/16 0.214
**FHIT gene**None/Either/Both p-value	23/26/13 0.237	30/29/3 0.334	21/30/11 0.899	–	24/24/14 0.088
**MGMT gene**None/Either/Both p-value	22/22/18 **0.024**	28/27/7 0.372	20/26/16 0.214	24/24/14 0.088	–

p value <0.05 was taken as significant.

**Table 5:** Comparison of clinical profile with the combinatorial effect of variations in genes in 62 patients with sporadic CRC.

Characteristic (N)	Novariation*n (%)	Any onevariation*n (%)	Any twovariations*n (%)	Any threevariations*n (%)	All fourvariations*n (%)	p-value
***Signs & Symptoms***						
Loss of weight (39)	5 (12.8)	9 (23.1)	9 (23.1)	9 (23.1)	7 (17.9)	**0.031**
Abdominal pain (35)	4 (11.4)	12 (34.3)	8 (22.9)	5 (14.3)	6 (17.1)	0.731
Bleeding per rectum (31)	4 (12.9)	11 (35.5)	9 (29)	5 (16.1)	2 (6.5)	0.614
Constipation (30)	7 (23.3)	8 (26.7)	8 (26.7)	4 (13.3)	3 (10)	0.389
Fatigue (28)	4 (14.3)	12 (42.8)	5 (17.9)	4 (14.3)	3 (10.7)	0.521
Obstruction features (24)	3 (12.5)	9 (37.5)	5 (20.8)	5 (20.8)	2 (8.3)	0.639
Loss of appetite (24)	3 (12.5)	9 (37.5)	5 (20.8)	5 (20.8)	2 (8.3)	0.639
Back pain (21)	3 (14.2)	5 (23.8)	6 (28.6)	1 (4.8)	6 (28.6)	0.063
Diarrhea (16)	3 (18.8)	6 (37.5)	3 (18.8)	3 (18.8)	1 (6.2)	0.759
Nausea & Vomiting (9)	1 (11.1)	3 (33.3)	2 (22.2)	1 (11.1)	2 (22.2)	0.920
Difficulty in micturition (6)	0 (0)	2 (33.3)	3 (50)	1 (16.7)	0 (0)	0.501
***Lifestyle factors***						
Fatty diet (32)	7 (21.9)	7 (21.9)	9 (28.1)	4 (12.5)	5 (15.6)	0.250
Non-vegetarian diet (30)	4 (13.3)	8 (26.7)	7 (23.3)	8 (26.7)	3 (10)	0.133
Spicy food (29)	6 (20.7)	5 (17.2)	8 (27.6)	4 (13.8)	6 (20.7)	0.096
Alcohol intake (28)	3 (10.7)	5 (17.9)	8 (28.6)	6 (21.4)	6 (21.4)	0.075
Smoking (26)	1 (3.8)	5 (19.2)	8 (30.8)	6 (23.1)	6 (23.1)	**0.016**
Lack of physical exercise (21)	2 (9.5)	7 (33.3)	7 (33.3)	4 (19.1)	1 (4.8)	0.504
Irregular intake of fruits (18)	0 (0)	5 (27.8)	8 (44.4)	5 (27.8)	0 (0)	**0.009**
Tobacco chewing (14)	2 (14.3)	5 (35.7)	2 (14.3)	3 (21.4)	2 (14.3)	0.805
***Tumor profile***						
Location: Colon (41)	5 (12.2)	13 (31.7)	10 (24.4)	7 (17.1)	6 (14.6)	0.900
Stage: 2, 3 & 4 (54)	6 (11.1)	16 (29.6)	15 (27.8)	9 (16.7)	8 (14.8)	**0.006**
Grade: MD, PD & UD (57)	9 (15.8)	18 (31.6)	16 (28.1)	7 (12.3)	7 (12.3)	0.662
Lymphatic invasion: Present (26)	2 (7.7)	4 (15.4)	8 (30.8)	5 (19.2)	7 (26.9)	**0.010**
Metastasis: Present (9)	0 (0)	1 (11.1)	1 (11.1)	4 (44.4)	3 (33.3)	**0.008**

* variation refers to genetic mutation in Kras gene and/or epigenetic modification in RASSF1A, FHIT and MGMT genes.

p value <0.05 was taken as significant.

## Discussion

The development of CRC is a multistep and widely studied model in tumorigenesis. Underlying changes at the genetic and/or epigenetic level may be involved in the overall development of CRC [4], [22]. Recent studies have focused on the mutations and methylation patterns of several genes implicated in the development of CRC. CpG island promoter methylation for transcriptional inactivation may occur at various stages of colon tumorigenesis, affecting various types of CRC to different degrees [23]. Identifying the interactions between the genetic and epigenetic alterations may aid in constructing personalized and specialized diagnostic molecular profiles and the development of newer therapeutic strategies [4]. To the best of our knowledge, this is the first study to elucidate the role of genetic mutation in Kras gene and epigenetic modification in RASSF1A, FHIT and MGMT genes in the development of adenocarcinomatous sporadic CRC in the Indian population.

Of the 62 patients included in the study, the most common signs and symptoms reported were loss of weight (63%), abdominal pain (57%) and bleeding per rectum (50%). Other studies have also reported bleeding in stool, abdominal pain, diarrhea or constipation as important signs and symptoms indicative of CRC [24].

The overall frequency of Kras mutation codon 12 and 13 was 47% and 19%, respectively. The mutation was also observed in 29% and 18% adjacent control regions at codon 12 and 13, respectively. This finding probably points out towards the fact that even though the adjacent mucosa was normal and non tumorous on pathology, the molecular changes in the form of mutation might have already been initiated in this region also. This could therefore be involved in the spread of the disease and hence Kras mutation may be considered as an early marker of identifying the spread of the disease into the adjacent areas. Studies worldwide have reported the frequency of Kras mutation in CRC to lie between 25–60% [25] and the most frequently observed types of mutations are G>A transitions and G>T transversions [6]. Kras mutation analysis has become increasingly important in CRC for indicating anti-EGFR antibody therapy as a predictive marker in CRC [26]. Clinical trials specifically focusing on this group are the emergent need of the hour.

Various studies have implicated the underlying factors and mechanisms in colorectal carcinogenesis and reported the involvement of lifestyle factors including dietary intake of fats, diet, dietary folate and alcohol intake in the development of CRC [5], [27], [28], [29]. High intake of dietary fat is also associated with an increased risk of mutated Kras colon tumors [5]. In the present study, however, no statistical association was observed and these findings are supported by others [30], [31]. Experimental studies suggest that by-products like malondialdehyde (MDA) and 4-hydroxynonenal (4-HNE) could be built up and stored due to peroxidation of v-6 polyunsaturated fatty acids (PUFAs) which in turn could react with DNA to form adducts like pyrimidopurinone adduct of deoxyguanosine (M1G), further resulting in mainly G>A and G>T transversions in bacteria [5]. It is noteworthy to mention here that the incidence of CRC is alarmingly increasing in India mainly due to changes in the lifestyle patterns. Urban style of living has witnessed the addition of various modifications in dietary patterns involving increased intake of junk foods. Homemade food is gradually being replaced by easy-to-prepare readymade foods. Fruits, vegetables and spices containing increased amounts of salicyclic acid have been shown to have an anticancer effect by sensitizing the normal cells to evade tumor formation [32]. However, adulteration in spices and increased use of chemicals in artificial ripening and growth of fruits and vegetables has decreased the anticancer potential of these foods. Magalhaes et al [33] have reported that the risk of colon cancer was increased with high intake of red and processed meat but no significant association was observed with rectal cancer. It has been suggested that G4A transitions at the second base of codon 12 or 13 of Kras gene in the human colon could be due to the N-nitroso compounds, present in processed meat or endogenously formed [34]. Heterocyclic aromatic amines (HAA) could be formed due to high temperature cooking of meat proteins whereas grilling and smoking of meat generates polycyclic aromatic hydrocarbons (PAH) as a result of fat dropping on open flames [28]. Shin et al have suggested the role of cigarette smoking and alcohol consumption in colorectal carcinogenesis. Cigarette smoking was found to confer a higher risk for advanced adenomas and 3 or more adenomas than for single risk or single adenomas [35]. Potential biological mechanisms for this association include the carcinogenic action of polynuclear aromatic hydrocarbons (PAHs), nitrosamines and aromatic amines, either present in, or produced by, burning tobacco [36]. Several ways have been suggested for the effect of alcohol on risk for CRC including acetaldehyde, an oxidation product of alcohol [37]. It has also been observed that microbially produced acetaldehyde from ethanol may increase the risk of colon cancer via folate deficiency [38]. Alcohol is an antagonist of methyl-group metabolism and may contribute to abnormal DNA methylation or may increase the risk for CRC indirectly through immune suppression, delay of DNA repair, activation of liver procarcinogens by induction of cytochrome P-450 enzymes, or changes in bile acid composition [39]. In the present study, alcohol intake and smoking habits were reported in 45% (28/62) and 42% (26/62) cases, respectively. The association was particularly evident in the case of male patients where 60% and 53% patients had these habits and is in agreement with the findings of Cho et al [40].

The promoter regions of several tumor suppressor genes contain CpG islands and may be involved in gene silencing by methylation [22]. RASSF1A promoter methylation was observed in 47% cases. Other authors have reported the frequency of RASSF1A mehylation between 16–81% cases in CRC [41], [42]. It has the potential to be an ideal cancer biomarker, occurring in a wide range of tumor types and comparatively not widely observed in the normal tissues [10]. RASSF1A methylation was also observed in only 8 cases in the normal mucosa suggesting that methylation event may have probably been initiated in the adjacent region also. Current and formers smokers have been shown to have a higher incidence of RASSF1A methylation and the incidence is even higher in tumors of a higher grade, later stage and more invasive or metastatic tumors [10]. In the present study, 42% patients had a history of smoking. Some studies have suggested an inverse correlation between RASSF1A methylation and Kras mutation in CRC [43]. It is not certain how RASSF1A affects its biological behavior despite their direct/indirect interaction with activated Ras. However, our study has shown a synergy between these two genes which may simply emphasize the importance of a host of genes involved in the signaling pathway.

FHIT promoter methylation was observed in 37% cases. FHIT promoter hypermehylation has been detected in 20–71% cases in CRC by only two authors [44], [45]. Sultana et al have suggested that FHIT gene may be involved in the etiology of various cancers other than cervical cancer, however, supporting data is scarce [46]. Although alternative splicing of human FHIT is not directly associated with carcinogenesis, FHIT may be inactivated frequently by exon skipping, intron retention, and activation of cryptic splice site within exon 6 in CRC [47]. Abnormal expression of the FHIT candidate tumor suppressor gene has been observed in a variety of human tumors, but little is known about its expression during colorectal tumorigenesis. A study from United Kingdom has shown reduced expression of FHIT in a small proportion of colonic precancerous lesions and in increased proportions of primary and metastatic colorectal cancers suggesting that FHIT plays a role in the development and progression of some colon carcinomas [48]. Our recent study has demonstrated novel missense mutation in FHIT gene and interpreted the effect in HPV-mediated cervical cancer in Indian women [49].

MGMT promoter methylation was observed in 47% cases in the present study. MGMT promoter hypermehylation has been detected in 20–41% cases in CRC [8], [25], [50]. Also, MGMT loss has been found to be associated with mutations in Kras [14]. Sidhu et al [13] have shown that the incidence of MGMT hypermethylation is significantly higher among subjects with history of smoking, alcoholism and intake of non-vegetarian food among prostate cancer cases, controls and subjects with benign prostatic hyperplasia. We observed that smoking (66%) and alcohol intake (62%) were the most common lifestyle factors observed in patients with MGMT hypermethylation. MGMT hypermethylation have been proven to be useful predictors of prognosis and responsiveness in gastric cancers and malignant gliomas [51], [52].

Brink et al [6] and Cejas et al [53] have not shown any statistical association between the tumor stage and metastasis with Kras mutation. However, our results are concordant with that observed by Mannan A [54] where they found that Kras mutations are significantly associated with lymph node metastasis and tumor stage but not with the growth pattern of colonic carcinomas. Kras mutations may be significantly involved in the biologic development of the disease, hence affecting its overall behavior and responsiveness.

Based on the results, the combinatorial presence of mutated Kras (codon 12) and methylated RASSF1A, FHIT and MGMT genes better characterize advanced tumor stage, metastatic disease, higher grade tumors and presence of lymphatic invasion than when considered separately. The conjunctional occurrence of more than one variation may act synergistically in affecting the aggressiveness of this disease and initiating tumor formation at these sites. These genetic and epigenetic variations work in unison in deciding the overall fate of the disease. It is not clear as to which occurs first, DNA hypermethylation or genetic mutation. In cancerous lesions, the barrier checking the spread of DNA methylation from the promoter region to the transcription site is abridged, therefore playing a critical role in tumor development, initiation and progression.

Comparisons in survival between the wild type/unmethylated group vs. mutant/methylated group were performed but were not statistically significant (p-value >0.05). This may be due to the shorter follow up data and the fact that only 1 event (death) occurred amongst the cohort of patients included in the study.

Taken together, the data demonstrated that tumor stage 3, moderately differentiated tumors, presence of lymphatic invasion and absence of metastasis were more frequently observed in tumors with mutated Kras and methylated RASSF1A, FHIT and MGMT genes. Hence, these markers may be used in assessing the overall pathological status of the disease for better targeting this heterogenous group of cancers. In conclusion, genetic mutation in the Kras gene and epigenetic modification in RASSF1A, FHIT and MGMT genes in sporadic CRC are associated with the overall development of the disease and may be used as diagnostic or prognostic markers in this group of cancers. The synergistic interrelationship between the genetic and epigenetic factors in colorectal tumorigenesis may help in improving the overall approach towards this disease.

## Supporting Information

Table S1 (DOC)
