# Supplementary material for: Kras Gene Mutation and RASSF1A, FHIT and MGMT Gene Promoter Hypermethylation: Indicators of Tumor Staging and Metastasis in Adenocarcinomatous Sporadic Colorectal Cancer in Indian Population
Source: PLoS One. 2013 Apr 3;8(4):e60142. doi: 10.1371/journal.pone.0060142 (PMC3616004; doi:10.1371/journal.pone.0060142)
Supplement: Table S1 — (DOC) [file pone.0060142.s001.doc]

**Table S1:** Biochemical profile of 62 patients with sporadic CRC

| **Characteristic** | **Kras gene**  **codon 12** | **Kras gene**  **codon 13** | **RASSF1A gene** | **FHIT gene** | **MGMT gene** |
| --- | --- | --- | --- | --- | --- |
|  | **Wild type (n=33)**  **Variant (n=29)**  **p-value** | **Wild type (n=50)**  **Variant (n=12)**  **p-value** | **UM* (n=33)**  **M* (n=29)**  **p-value** | **UM* (n=39)**  **M* (n=23)**  **p-value** | **UM* (n=33)**  **M* (n=29)**  **p-value** |
| Blood glucose (mg/dl) | 98.9±30.7  105.9±30.9  0.374 | 100.1±25.2  110.4±48.1  0.303 | 95.8±33.1  109.4±26.7  0.082 | 94.6±30.6  115±27  **0.010** | 97.9±30  106.9±31.4  0.255 |
| SGPT^#^  (U/L) | 20.5±10.3  16.6±5.4  0.077 | 19.3±9.1  16.7±5.7  0.353 | 19.6±9.1  17.8±8.1  0.440 | 19.7±9.5  17±6.6  0.235 | 19.4±9.4  17.9±7.7  0.476 |
| SGOT^##^  (U/L) | 23.7±6.8  22.6±7.9  0.077 | 25.2±6.3  22.7±7.5  0.302 | 23.9±7.4  22.6±7.3  0.491 | 24.3±7  21.1±7.7  0.111 | 23.5±7.2  22.8±7.6  0.715 |
| ALP^###^  (U/L) | 114.6±71.3  163.5±102.4  **0.033** | 133.3±79.1  156.4±128.6  0.431 | 134±74.5  142.1±105.9  0.730 | 133.4±93.8  145.3±23  0.620 | 126.9±87.7  149.9±9208  0.323 |
| Total bilirubin  (mg/dl) | 0.9±1.8  0.6±0.26  0.354 | 0.8±1.4  0.6±0.2  0.617 | 0.9±1.8  0.7±0.3  0.546 | 0.9±1.6  0.6±0.3  0.453 | 0.9±1.7  0.6±0.2  0.402 |
| Direct bilirubin  (mg/dl) | 0.2±0.1  0.2±0.1  0.107 | 0.2±0.1  0.2±0.1  0.701 | 0.2±0.1  0.2±0.1  0.060 | 0.2±0.1  0.2±0.1  0.933 | 0.2±0.1  0.2±0.1  0.543 |
| Total protein  (gm/dl) | 6.9±0.9  6.9±0.7  0.714 | 6.6±0.9  7±0.8  0.158 | 6.7±0.8  7.1±0.8  **0.038** | 6.8±0.9  7.2±0.6  0.050 | 6.9±0.9  7±0.7  0.531 |
| Albumin  (gm/dl) | 3.8±0.4  3.9±0.6  0.726 | 3.9±0.6  3.5±0.8  0.071 | 3.9±0.7  3.7±0.7  0.108 | 3.7±0.7  3.9±0.6  0.151 | 3.8±0.7  3.9±0.7  0.522 |
| Globulin  (gm/dl) | 3.2±0.5  3±0.6  0.396 | 3.1±0.5  3.1±0.8  0.719 | 3.1±0.4  3±0.6  0.491 | 3±0.5  3.2±0.5  0.279 | 3.1±0.5  3.1±0.5  0.906 |
| GGT^####^  (U/L) | 20.9±11.3  37.2±45.5  0.076 | 27.5±33.5  34.3±34.8  0.556 | 29.1±39.9  28.6±26.9  0.955 | 20.7±14  42.4±49.4  **0.021** | 27.7±39.4  30.3±25.4  0.778 |
| Urea  (mg/dl) | 23.7±9.5  22.1±8.9  0.516 | 22.9±7.9  23±13.5  0.995 | 23.1±8.9  22.8±9.6  0.901 | 24.2±9.8  20.9±7.8  0.182 | 23.5±9  22.4±9.5  0.629 |
| Creatinine  (mg/dl) | 0.9±0.2  0.8±0.2  0.169 | 0.9±0.2  0.8±0.2  0.867 | 0.8±0.2  0.8±0.2  0.435 | 0.8±0.2  0.8±0.2  0.672 | 0.8±0.2  0.8±0.2  0.404 |
| Uric acid  (mg/dl) | 4.9±1.3  4.8±1.7  0.729 | 5±1.5  4.2±1.3  0.108 | 5.1±1.6  4.6±1.3  0.286 | 4.8±1.4  4.9±1.7  **0.042** | 4.8±1.5  4.9±1.5  0.726 |

* UM, unmethylated; M, methylated; ^#^ SGPT, serum glutamic pyruvic transaminase; ^##^ SGOT, serum glutamic oxaloacetic transaminase; ^###^ ALP, Alkaline phosphatase; ^####^ GGT, gamma-glutamyl transpeptidase

values expressed as mean ± standard deviation, p value < 0.05 was taken as significant
